# Supplementary material for: ctDNA monitoring using tumor-informed copy number analysis
Source: EMBO Mol Med. 2026 Mar 19;18(4):1429–55. doi: 10.1038/s44321-026-00399-4 (PMC13083984; doi:10.1038/s44321-026-00399-4)
Supplement: Supplementary file 9 — Expanded View Figures [file 44321_2026_399_MOESM9_ESM.pdf]

Expanded View Figures

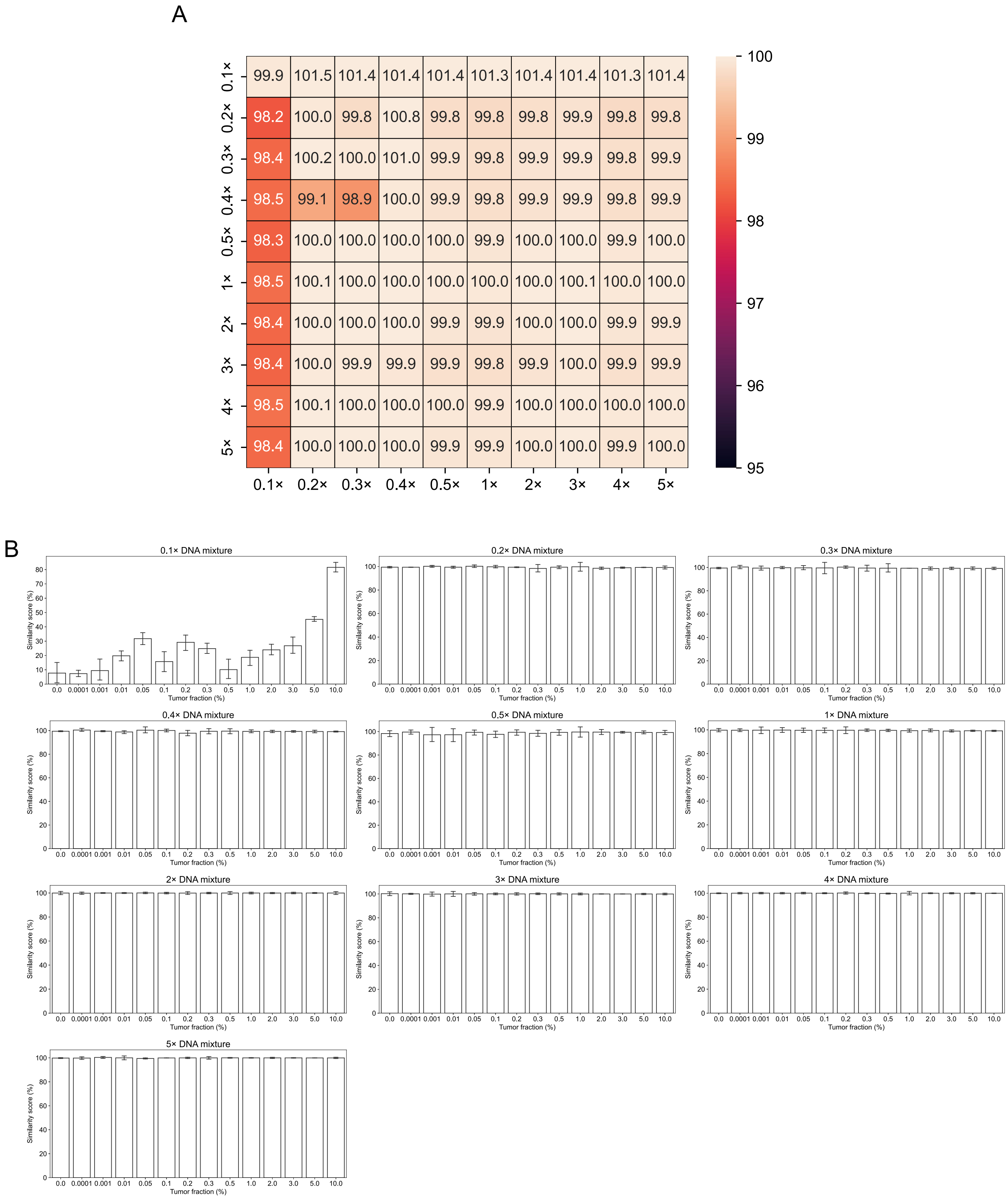

 © The Author(s)

◀ **Figure EV1. Robustness and reproducibility assessment of copy number similarity scores in cancer cell line data.**

(**A**) Average copy number Similarity Scores across different down-sampling depths in sWGS samples from the same cancer cell line (tumor) data for robustness assessment. Similarity Scores exceeding 100% indicate that the query sample is considered to have a higher TF estimate than the reference sample. (**B**) Copy number Similarity Scores between replicates with the same tumor fraction and sequencing depth in the dilution series for reproducibility assessment, with error bars indicate 95% confidence intervals. In these pairwise comparisons, each replicate, in turn, served as the reference for comparison with the other replicates, resulting in a total of 60 comparisons per condition.

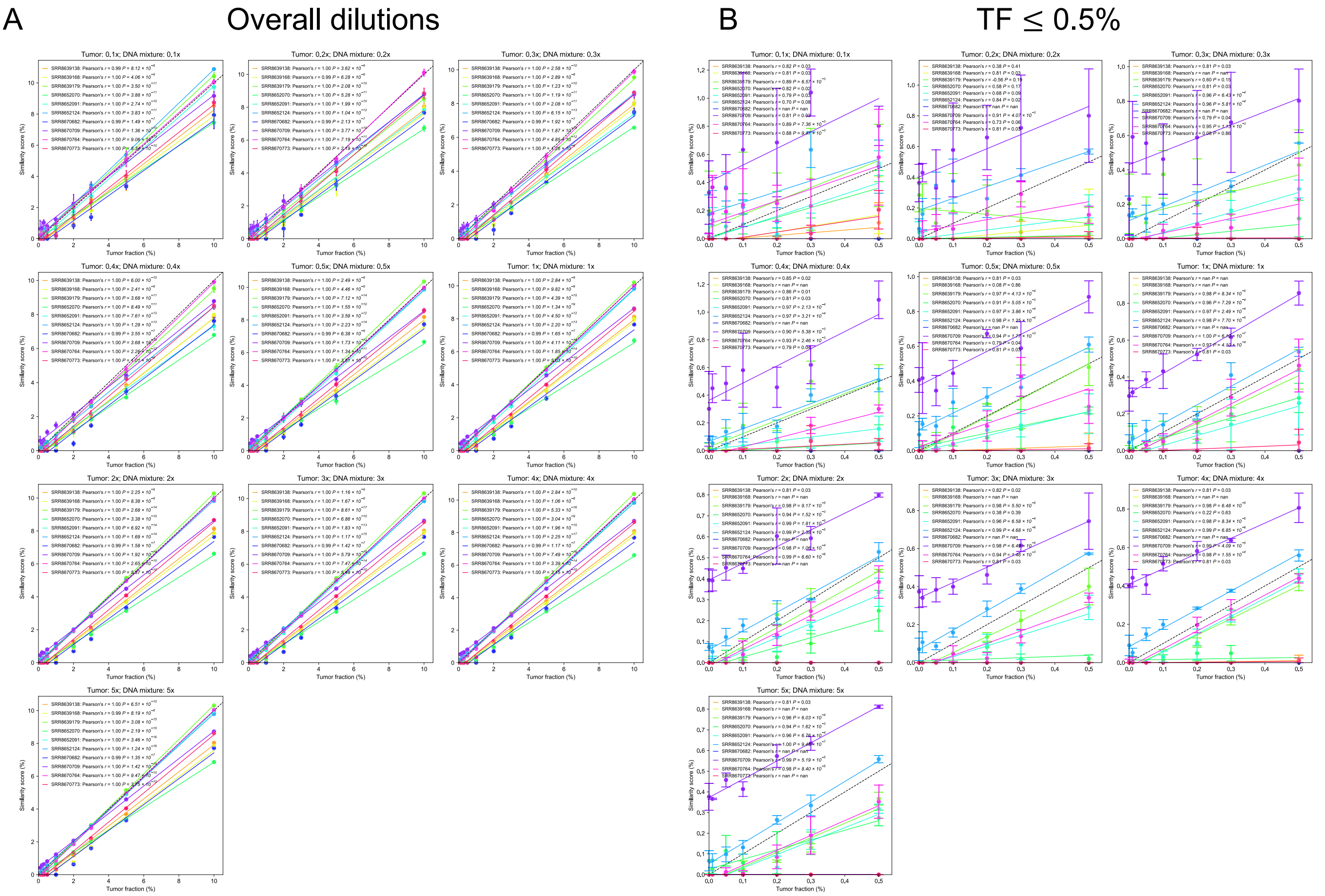

Figure EV2. Correlations between tumor DNA fractions (TF) and copy number Similarity Scores in diluted cancer cell line samples.

Error bars show 95% confidence intervals of three replicates for each TF condition. (**A**) Overall tumor fractions. (**B**) Samples with tumor DNA fractions ≤ 0.5%.

 © The Author(s)

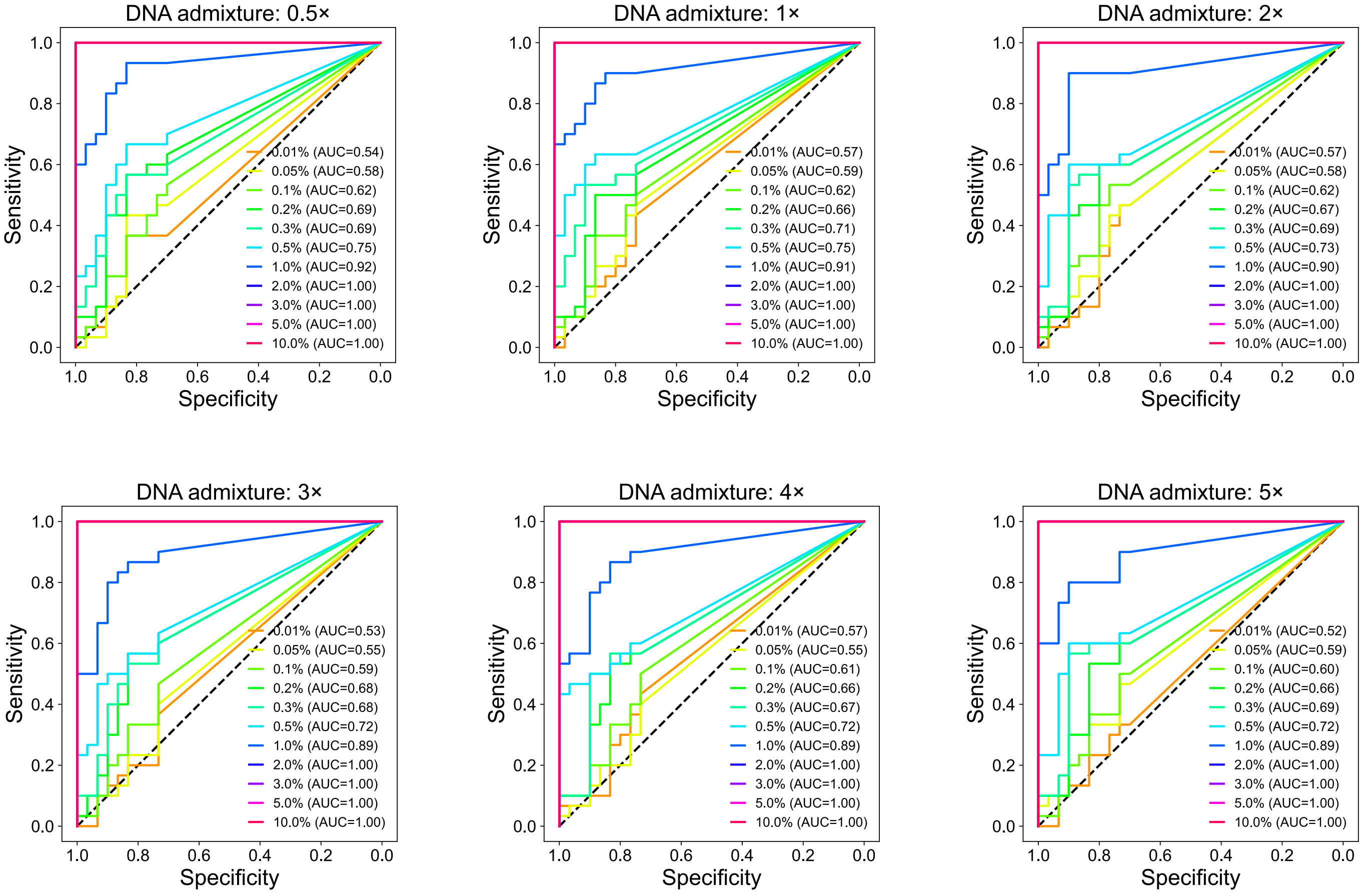

Figure EV3. Performance of tumor DNA detection across sequencing depths and tumor fractions.

ROC curves for differentiating diluted WGS data with and without tumor DNA across different sequencing depths and tumor fractions.

© The Author(s)

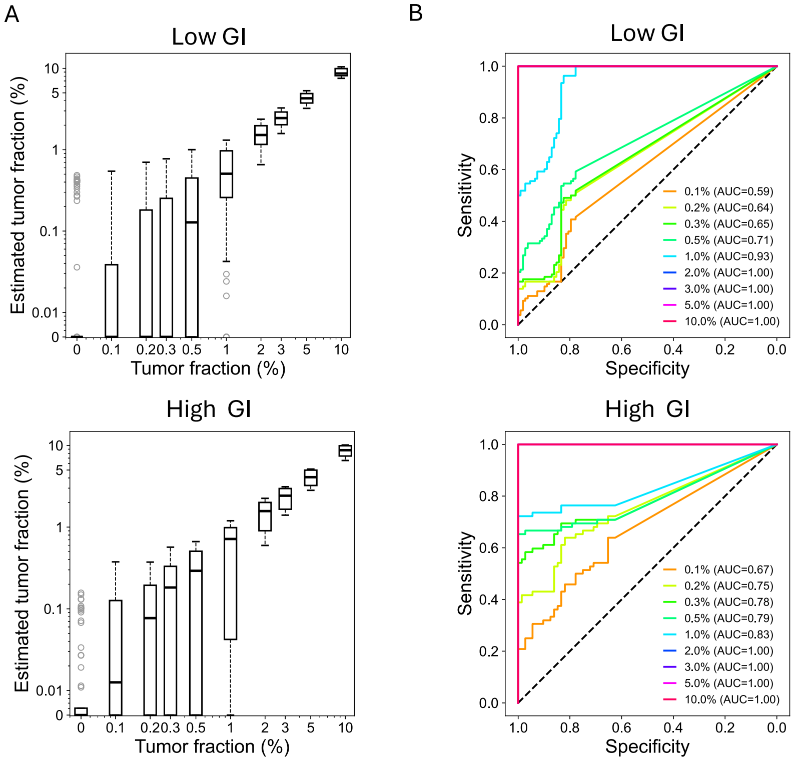

C

Low GI

TF (%):	0.1	0.2	0.3	0.5	1	2	3	5	10
Sensitivity at 95% specificity	10%	15%	17%	30%	55%	100%	100%	100%	100%
Sensitivity at 90% specificity	13%	16%	18%	33%	59%	100%	100%	100%	100%
Sensitivity at 85% specificity	16%	18%	23%	45%	72%	100%	100%	100%	100%
Sensitivity at 80% specificity	36%	48%	49%	54%	96%	100%	100%	100%	100%

High GI

TF (%):	0.1	0.2	0.3	0.5	1	2	3	5	10
Sensitivity at 95% specificity	25%	41%	58%	65%	72%	100%	100%	100%	100%
Sensitivity at 90% specificity	30%	43%	59%	66%	72%	100%	100%	100%	100%
Sensitivity at 85% specificity	36%	53%	65%	66%	74%	100%	100%	100%	100%
Sensitivity at 80% specificity	43%	61%	69%	68%	74%	100%	100%	100%	100%

Figure EV4. Analyses of in silico cancer cell line DNA dilution series with cancer types with high and low genomic instability (GI).

The high GI group included four cell lines, including MKN-45 (gastric adenocarcinoma), KNS62 (non-small cell lung carcinoma), RMG-I (ovarian carcinoma), and SK-MEL-24 (melanoma). The remaining six cell lines were grouped into the low GI category, including AN3-CA (endometrial adenocarcinoma), H4 (glioma), LAMA-84 (chronic myeloid leukemia), SK-N-AS (neuroblastoma), SUIT-2 (pancreatic ductal adenocarcinoma), and 22Rv1 (prostate adenocarcinoma). (**A**) Boxplot of informCNA estimated TFs, with 72 and 108 estimations per tumor fraction in the high and low GI groups, respectively. The central line representing the median, the bounds of box indicating the interquartile range (IQR; 25% and 75% percentiles), and whiskers extend to the most extreme values within 1.5 times IQR. Outliers exceeding the whiskers are plotted individually to represent the full data range, including minimum and maximum values. (**B**) ROC curves for tumor DNA detection using informCNA estimated TF. (**C**) Sensitivity of informCNA tumor detection across different specificities.

© The Author(s)

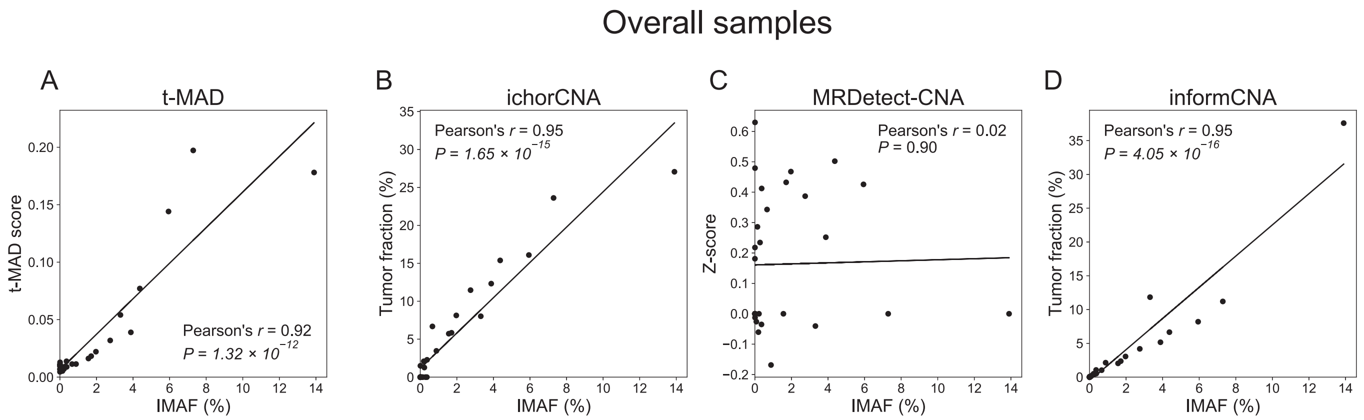

Figure EV5. Comparison of IMAF with copy-number–based ctDNA metrics.

Correlation between IMAF values and the outputs of tMAD (**A**), ichorCNA (**B**), MRDetect-CNA (**C**), and informCNA (**D**) across all time points in patients with breast cancer.

© The Author(s)

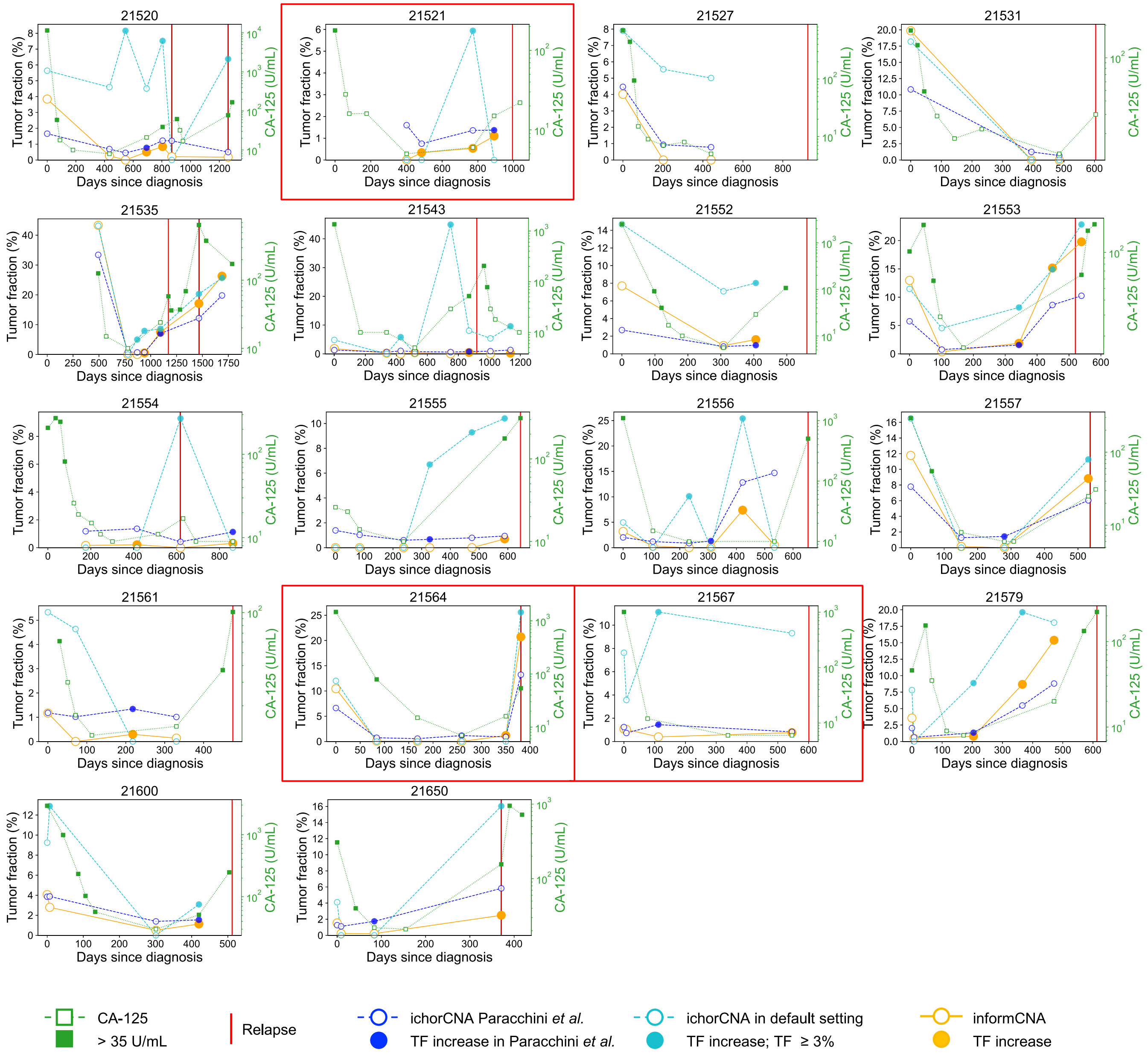

Figure EV6. Performance of different assays in longitudinal monitoring of ovarian cancer patients undergoing chemotherapy.

CA-125 levels (open green square) > 35 U/mL (filled green square) indicate detected recurrence. ctDNA tumor fractions (TFs) in plasma cfDNA are estimated either by ichorCNA in default setting (cyan circle) or with manual curation (blue circle; Paracchini et al), and by informCNA (yellow circle). In patient 21521, the manually curated ichorCNA TF at the second plasma time point showed a decreasing trend, whereas both CA-125 levels and informCNA estimated TF showed an increasing trend. In patient 21564, at the fourth plasma time point, the manually curated ichorCNA TF showed an increase, while both CA-125 and informCNA TF indicated a decrease. For patient 21567, both the manually curated and default ichorCNA TFs showed an increasing trend, in contrast to the decreasing trends observed in CA-125 and informCNA estimated TF. Notably, manually curated ichorCNA results also differed from those obtained using the default settings.

 © The Author(s)

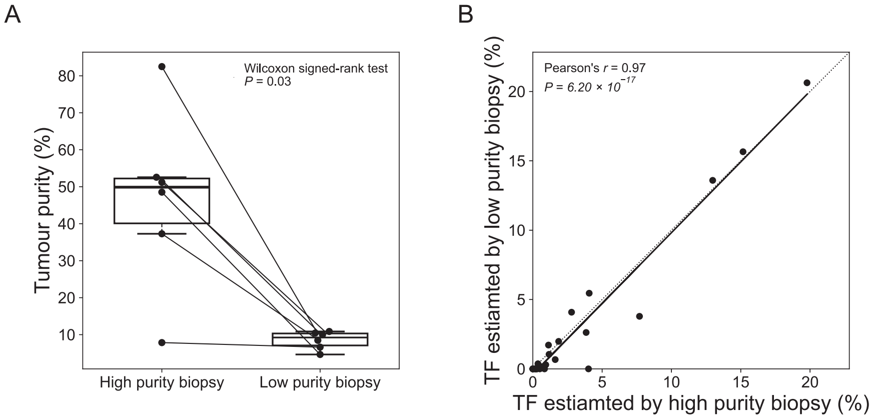

Figure EV7. ctDNA detection using references with low tumor purity.

(**A**) Tumor purity of low-purity biopsies (median: 9.2%; range: 4.6 to 10.9%) and their high-purity compartments (median: 49.9%; range: 7.8 to 82.5%). The central line representing the median, the bounds of box indicating the interquartile range (IQR; 25% and 75% percentiles), and whiskers extend to the most extreme values within 1.5 times IQR. Outliers exceeding the whiskers are plotted individually to represent the full data range, including minimum and maximum values. (**B**) Correlation between TFs estimated by informCNA using either the high- or low-purity biopsy as the reference sample in same patient.

© The Author(s)

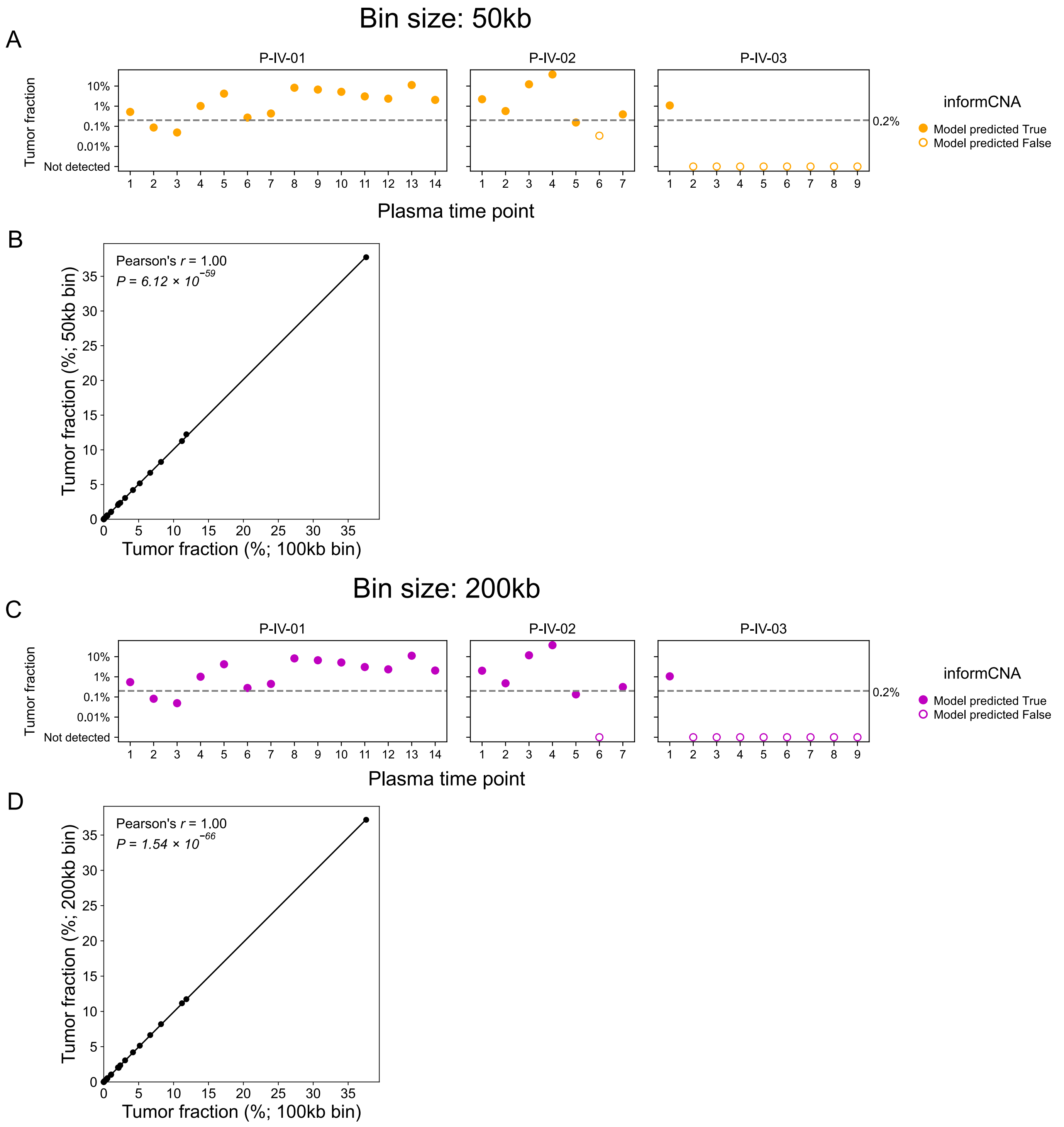

Figure EV8. ctDNA detection and tumor fraction correlation analyses using different window sizes.

(**A**) Tumor fraction estimated by informCNA using 50 kb bins. (**B**) Correlation between tumor fractions estimated by using 100 kb and 50 kb bins. (**C**) Tumor fraction estimated by informCNA using 200 kb bins. (**D**) Correlation between tumor fractions estimated by using 100 kb and 200 kb bins.

 © The Author(s)

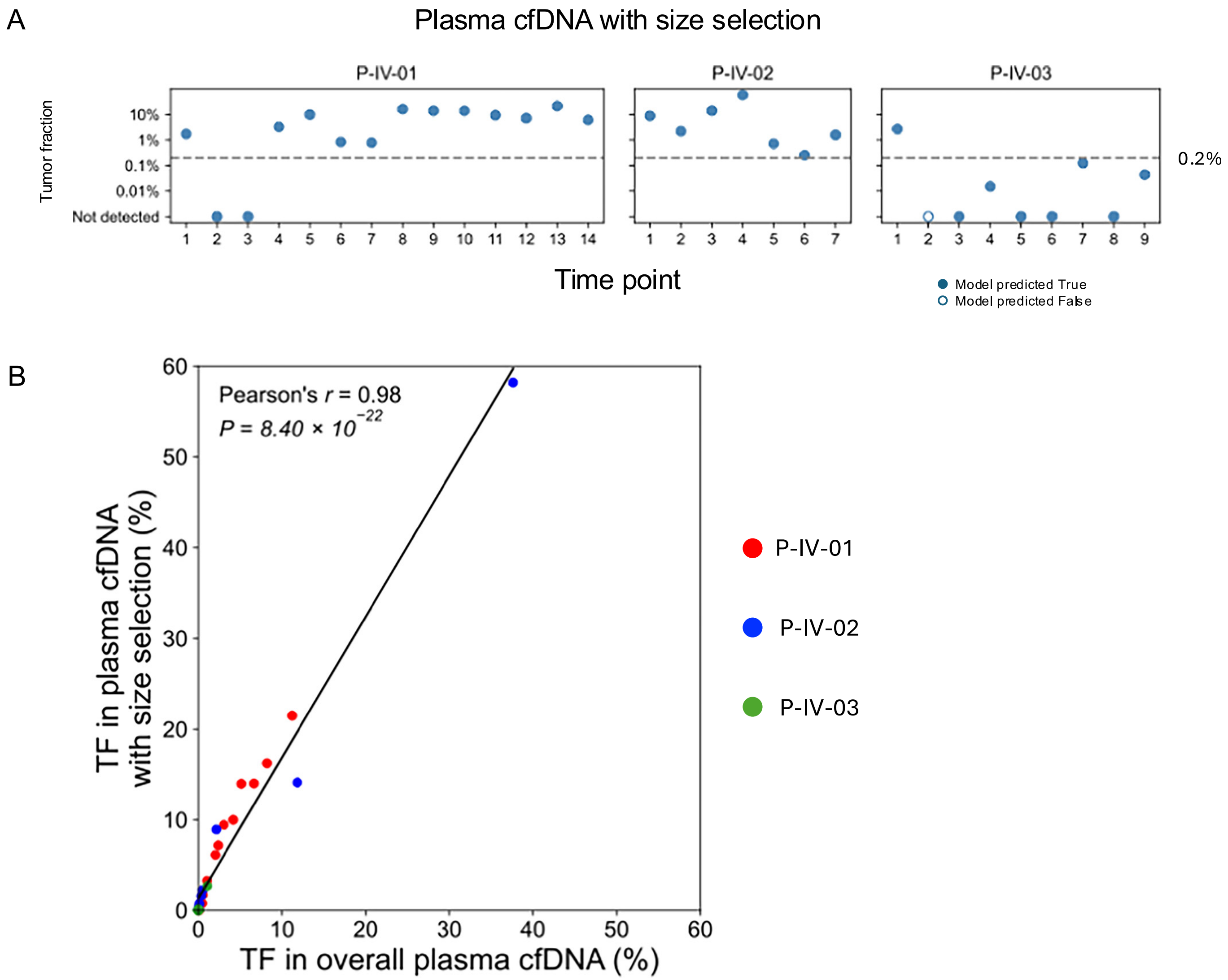

Figure EV9. ctDNA detection and tumor fraction estimation using in silico size selection.

(**A**) Tumor fraction estimated by informCNA using in silico size selection of short plasma cfDNA (< 150 bp). (**B**) Correlation between tumor fractions estimated from overall plasma cfDNA without size selection and from in silico size selected short cfDNA.
